# Urgent Direct Access to Diagnostic Services for General Practitioners: Bridging the Gap in Cancer Diagnosis

**DOI:** 10.7759/cureus.63350

**Published:** 2024-06-28

**Authors:** Roji Gurung, Anna Podlasek

**Affiliations:** 1 Radiology, Nottingham University Hospital, Nottingham, GBR; 2 Radiological Sciences, University of Nottingham, Nottingham, GBR; 3 Radiology and Imaging Technology, University of Dundee, Dundee, GBR

**Keywords:** radiology, general practice, ct chest, us abdomen and chest, ct abdomen and chest, urgent direct access tests, mri brain, chest-x-ray (cxr), cancers

## Abstract

Urgent direct access to diagnostic services for general practitioners (GPs) is a new pathway to capture any cancer diagnoses that may have been missed due to vague symptom presentations. Hence, GPs should look out for the key symptoms mentioned by NHS England that should prompt urgent direct access referrals for chest X-ray (CXR), computed tomography (CT) chest, MRI brain, ultrasound (US) abdomen and pelvis, and CT abdomen and pelvis. By implementing this approach, we can significantly reduce the time to diagnosis, while minimizing the number of visits to GP and specialist appointments prior to initiating investigations. However, the use of this pathway can only improve if access to diagnostic scans is improved. This needs to be done by ensuring all GPs in the country have access to directly request MRI brains, CT chest, abdomen, and pelvis. Further research into the impact of the urgent direct access pathway as well as investigating the number of GPs without access to these vital diagnostic services is required to fully improve and measure the progress of this referral pathway.

## Introduction and background

Over one in five cancer diagnoses are made in people referred for investigations on non-urgent pathways - usually because their vague symptoms did not fall into pre-specified symptoms criteria for a particular cancer pathway [1]. Therefore, it is vital these patients are not overlooked to improve timely diagnosis. Patients with delays in their cancer diagnosis can wait longer than eight weeks to be diagnosed [2]. Recognizing the urgency, NHS England has implemented urgent direct access imaging tests, available for general practitioners (GPs) to request since November 2022. The primary objective of these tests is to expedite the diagnostic process and reduce numerous GP and specialist appointments prior to initiating investigations [3]. This initiative is aligned with the NHS Long Term Plan, aspiring to achieve early diagnoses for 75% of cancer patients by 2028 [4]. Furthermore, it aims to ensure that 95% of patients requiring a diagnostic test, receive it within six weeks by 2025 [5].

The urgent direct access imaging pathway aims to complete image acquisition and reporting within four weeks from GP referral. This is in line with NHS England's new Faster Diagnosis standard which has replaced the two-week wait timeframe for urgent cancer referrals to diagnosis or ruling out of cancer within 28 days of referral [6]. Given more capacity required for this pathway, NHS England had planned to open community diagnostic centers for a full seven days a week and improve digital connectivity to meet this target time [7]. That would especially benefit around 67,000 people who are usually diagnosed with cancer through non-urgent testing - that now would be eligible for fast-tracking - and can have a better chance of having their disease detected at an earlier stage with higher survival chances [8]. Therefore, this is a review of NHS England’s guidance for urgent direct access imaging referral for GPs and the symptoms that qualify for each diagnostic test.

## Review

The three urgent referral pathways for cancer

There are three urgent referral pathways that can be utilized by GPs for cancer diagnoses. These include site-specific suspected urgent cancer referral, non-specific symptoms referral, and urgent GP direct access referral. NHS England has deemed urgent cancer referrals as those that are high-risk with specific symptoms that meet the threshold in the National Institute for Health and Care Excellence (NICE) cancer referral guidelines. Non-specific symptoms referral includes high risk with non-specific symptoms that could indicate more than one type of cancer. Finally, urgent direct access referral includes low risk with specific symptoms that do not meet the thresholds of the NICE cancer referral guidelines [3]. It is for the final referral pathway that GPs can directly request urgent diagnostic imaging to capture any vague cancer presentations.

**Figure 1:**
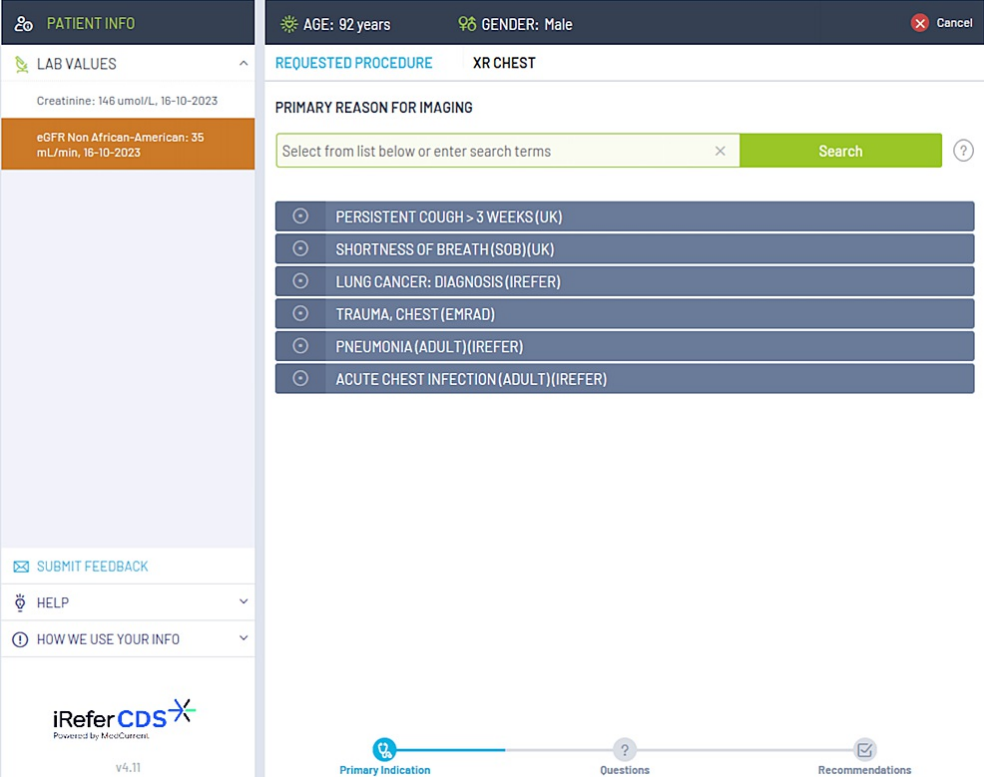
An example of how the iRefer clinical decision support system suggests the correct imaging modality based on the clinical picture, for example, how various chest symptoms might prompt a chest X-ray.

**Figure 2:**
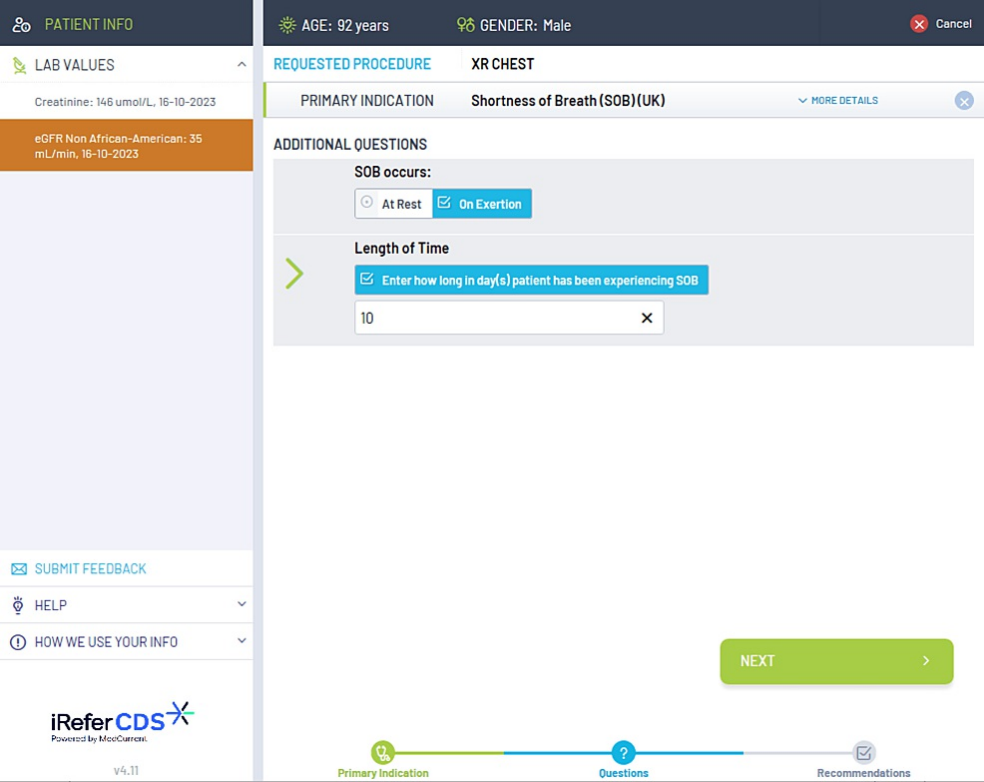
Exemplifies, if shortness of breath is selected, how the iRefer clinical decision support system asks additional questions to clarify which imaging modality is appropriate.

**Figure 3:**
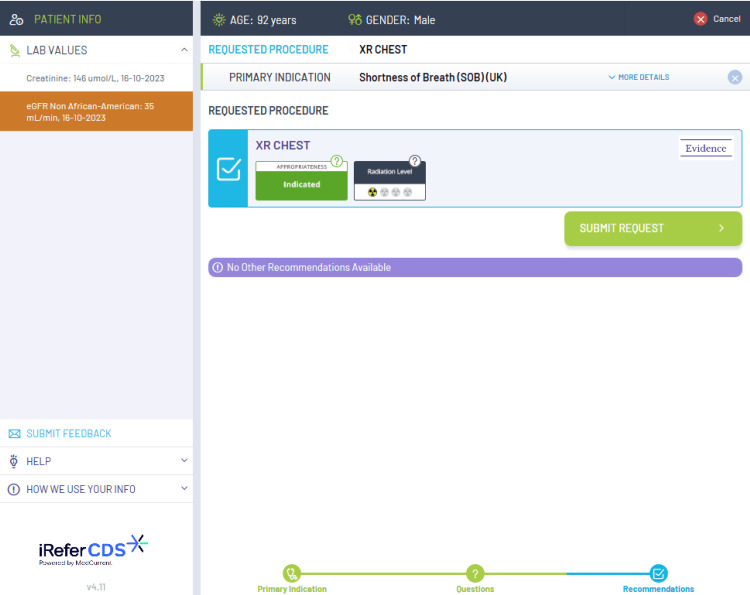
The iRefer clinical decision support system suggesting the most appropriate imaging modality based on the answers to previous questions.

The current diagnostic services include a range of imaging modalities, including chest X-ray (CXR), CT chest, CT abdomen and pelvis, ultrasound (US) abdomen and pelvis, and brain MRI. NHS England states that all GPs should have access to these imaging modalities as a minimum [3]. The choice of which imaging modality should be consulted is based on the iRefer clinical decision support (CDS) guidelines, which are evidence-based guidelines released by The Royal College of Radiologists. These guidelines ensure that the correct imaging modality is selected from the clinical picture which helps to support rapid diagnosis and avoids unnecessary ionizing radiation [9]. The iRefer CDS guidelines are embedded into the electronic request system such as the Sunquest ICE system, however, there is no current information on its availability in the general practice across the country. Figures 1, 2, 3 show examples of how the iRefer CDS within the electronic requesting system selects the appropriate imaging modality based on the symptoms of the patient.

CXR and CT chest

Lung cancer is responsible for 6% of annual mortality in the UK [10]. Although CXR is a low-cost and time-efficient scan, its sensitivity to detect lung cancer is only 78-80% [11]. Around 90% of missed lung cancer cases occur on CXR, and around 5% occur on CT chest, making CT chest more accurate for lung cancer screening. Given CTs can view images in three-dimensional planes, allowing for greater resolution in the picture, thus enabling better lung visualization [12]. However, the limitations of CTs include higher costs for imaging and reporting with CTs and 400% greater radiation exposure than CXR [13]. Therefore, CXR should be reserved for patients who are deemed lower risk of lung cancer including never-smokers and patients less than 40 years old. NHS England suggests the symptoms that should prompt for CT chest include patients with unexplained persistent cough of greater than three weeks where normal CXR would not be reassuring, worsening spirometry, any cervical lymphadenopathy, and continued undiagnosed symptoms despite normal CXR [3]. These symptoms, seen in Table 1, are all deemed lower risk and suitable for the urgent direct access pathway. It differs from the urgent cancer referral criteria for lung cancer, as seen in Table 2 [14].

**Table 1:** The symptoms that should prompt an urgent direct access request for the various imaging modalities by the general practitioner. CXR: chest X-ray; US: ultrasound

Imaging modality	Symptoms that should prompt urgent direct access tests
CT chest	Unexplained persistent cough of >3 weeks where normal CXR would not be reassuring. Worsening spirometry. Cervical lymphadenopathy. Continue undiagnosed symptoms despite normal CXR.
Chest X-ray	Symptoms as CT chest but patients who are: never-smokers. Less than 40 years old.
Brain MRI	New persistent or progressive headache with the following characteristics: cognitive changes. Speech changes. Personality changes. Objective vision changes. Unilateral limb weakness. Unilateral sensory changes.
US abdomen and pelvis or CT abdomen and pelvis	Pelvic or abdominal pain. Persistent abdominal bloating/distension. Increased urinary urgency +/- frequency. New onset altered bowel habit in >40 years old. Early satiety +/- loss of appetite.

**Table 2:** The National Institute for Health and Care Excellence (NICE) criteria of symptoms for urgent cancer referral for suspected lung cancer.

Urgent cancer referral for lung cancer	Symptom criteria
Urgent cancer referral for an appointment	CXR findings suggestive of lung cancer. >40 years old with unexplained hemoptysis.
Urgent cancer referral for chest X-ray	>40 years old with unexplained: persistent/recurrent chest infections. Finger clubbing. Supraclavicular lymphadenopathy or persistent cervical lymphadenopathy. Chest signs consistent with lung cancer. Thrombocytosis. >40 years old with two or more or if they have ever smoked with one or more of the following unexplained symptoms: Cough. Fatigue. Shortness of breath. Chest pain. Weight loss. Appetite loss.

Brain MRI

There are around 9000 new primary brain cancers diagnosed each year in the UK [15]. The majority of brain cancer diagnoses (50.1%) are diagnosed via accident and emergency (A&E) presentation and urgent cancer referral pathways only find 3.3% of brain tumors [16]. The rest are diagnosed via routine or urgent GP referrals not done via the urgent cancer referral pathway as well as both inpatient and outpatient routes [17]. NICE suggests that progressive and subacute loss of central neurological function is an indication for urgent cancer referral [15]. Brain MRI can be referred under the urgent direct access pathway if there have been any changes in the frequency and severity of headaches. The symptoms that should prompt referral include a new persistent or progressive headache with the following characteristics: cognitive changes, changes in speech, personality changes, objective changes in vision, unilateral limb weakness, or unilateral sensory changes [3].

Although direct access brain MRI is recommended by NHS England, some GPs still do not have direct access to MRI requests. Hence, patients with the above symptoms end up being referred via the urgent cancer referral pathway instead where a brain MRI can be requested by secondary care. However, which specialty is being referred to varies with local pathway guidelines. Audits of neurosurgery found that they were not the most appropriate specialists to refer to given the high rates of normal imaging or other diseases found where specialties such as neurology are best suited [16]. Therefore, although the referral pathway for urgent direct access to brain MRI exists, more resources are required to improve overall access to brain MRIs for all GPs.

US abdomen and pelvis and CT abdomen and pelvis

There are several intra-abdominal or pelvic cancers that can be diagnosed with US or CT abdomen and pelvis. These include lymphomas, esophageal, stomach, pancreatic, and colorectal cancers where CT is primarily used, and urological and gynecological cancers for which US can also be used. The intra-abdominal and pelvic cancers account for 47% of all cancer in the UK and 46% of mortality of all cancers as well [18]. Hence, it is imperative to identify and refer these cancers in a timely manner.

The symptoms that qualify for the urgent direct access pathway include pelvic or abdominal pain, persistent abdominal distension, or bloating, increased urinary urgency +/- frequency, new onset altered bowel habit in those above 40 years old, and early satiety +/- loss of appetite. NHS England suggests that the choice between US abdomen and pelvis and CT abdomen and pelvis for the lower risk symptoms should be decided based on the iRefer-CDS tool [3].

**Figure 4:**
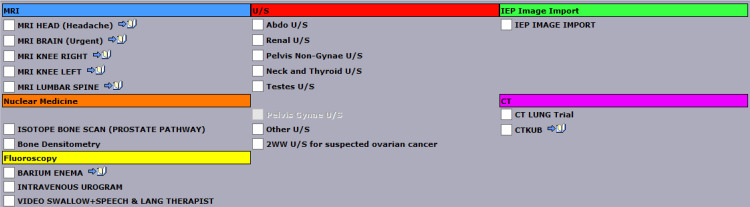
All the scans (except for plain film X-rays) available for direct access scan in a General Practice in Nottingham as of 6th of November 2023.

Similar to brain MRI, not all GPs have access to request CT abdomen and pelvis. Figure 4 shows the available scans excluding plain film X-rays that can be ordered by a GP in Nottingham with no CT abdomen and pelvis on the list. CT abdomen and pelvis are considered to cause one of the highest exposures to radiation with a lifetime additional risk of fatal cancer per examination as one in 2000, whereas a CXR causes 1 in 1,000,000 [19]. However, it is important to note that the US are dynamic scans that might have a limited view when an overlying bowel or gas obstructs the view of underlying organs and these studies are operator-dependent. Hence, the importance of each imaging procedure is to be justified and optimized with the lowest radiation dose possible to get an accurate diagnostic CT scan [20]. This is where the iRefer-CDS tool can greatly help determine whether a US scan answers the clinical questions instead. The requests are also reviewed by radiologists in detail for the indication and to compare the potential benefit with the potential radiation hazard or unnecessary load on resources to finalize which scan is best for the patient.

How to improve communication between primary care and radiology?

To improve the referral communication between primary care and radiology, NHS England suggests that GPs should ask a specific clinical question on the request form that the imaging is required to answer. It should detail the exact symptoms and their location, any relevant past medical history, or tests, and the suspected condition the imaging is looking for. The information in the radiology report should include an unambiguous specific clinical answer tailored to the professional background of the referrer. It should include key findings of the report, the next actionable steps, and any suggestions for further specialist review or investigations required. Asking the appropriate clinical questions and receiving a prompt and actionable clinical answer should improve the efficiency of urgent direct access referrals [3].

Limitations of the urgent GP direct access referral pathway

The provision of direct access for GPs to diagnostic services for patients with symptoms that do not meet the threshold for urgent suspected cancer referral has the potential for increased demand for diagnostic services, which could lead to longer waiting times and resource strain. The current radiology workforce shortage needs to be taken into consideration [21].

Another disadvantage is the risk of overdiagnosis and overtreatment. Providing broader access to diagnostic tests may lead to the identification of abnormalities that would never cause symptoms or death (incidentalomas), leading to unnecessary further tests and treatments that carry their own risks and costs. This could cause psychological stress for patients and may lead to more invasive procedures that are not clinically justified [22].

Moreover, there may be variability in GP expertise and judgment in utilizing direct access services, which can result in inconsistent use of diagnostic resources. Without stringent criteria or adequate training, there could be discrepancies in how and when diagnostics are employed, potentially leading to underuse in some cases and overuse in others. It will also add a significant workload burden in primary care [23]. Moreover, the lack of immediate specialist input burden may cause potential delays in diagnosis and management by GPs.

## Conclusions

Urgent direct access to diagnostic services for GPs is vital to capture any cancer diagnoses that may have been missed due to vague symptom presentations. Hence, GPs should look out for the key symptoms as mentioned by NHS England that should prompt urgent direct access referrals. However, the use of this pathway can only improve if access to diagnostic scans improves. This needs to be done by ensuring all GPs in the country have access to directly request MRI brains, CT abdomen and pelvis, and CT chest. Further research into the impact of the urgent direct access pathway as well as investigating the number of GPs without access to these vital diagnostic services is required to fully improve and measure the progress of this referral pathway.
